# Uncorrected Dextro–Transposition of the Great Arteries With Intact Ventricular Septum and Atrial Septal Defect Diagnosed After Developing Heart Failure

**DOI:** 10.1016/j.cjcpc.2025.06.003

**Published:** 2025-06-27

**Authors:** Kohsaku Goto, Akihito Saito, Hiroyuki Tokiwa, Masahiko Umei, Katsura Soma, Imari Mimura, Ryo Inuzuka, Yasutaka Hirata, Koichiro Niwa, Norihiko Takeda, Atsushi Yao

**Affiliations:** a Department of Cardiovascular Medicine, Graduate School of Medicine, the University of Tokyo, Tokyo, Japan; b Department of Computational Diagnostic Radiology and Preventive Medicine, the University of Tokyo Hospital, Tokyo, Japan; c Division of Nephrology and Endocrinology, Graduate School of Medicine, the University of Tokyo, Tokyo, Japan; d Organ Pathophysiology Program, Graduate School of Medicine, the University of Tokyo, Tokyo, Japan; e Department of Pediatrics, the University of Tokyo Hospital, Tokyo, Japan; f Department of Cardiovascular Surgery, the University of Tokyo Hospital, Tokyo, Japan; g Division of Cardiovascular Surgery, National Center for Child Health and Development, Tokyo, Japan; h Department of Cardiology, St Luke’s International Hospital, Tokyo, Japan; i Division for Health Service Promotion, the University of Tokyo, Tokyo, Japan

**Keywords:** congenital heart disease, heart failure, complete transposition of the great arteries, pulmonary artery aneurysm

**Dextro–transposition of the great arteries (d-TGA) with TGA with atrioventricular concordance and ventriculoarterial discordance is a major cyanotic congenital heart disease, and patients with d-TGA who do not undergo surgical correction are generally unlikely to survive into adulthood. Here, we present a female patient with uncorrected d-TGA with an intact ventricular septum and an atrial septal defect who survived for 69 years. She was diagnosed at the age of 57 years, when she was hospitalized for heart failure. Thereafter, she was followed closely for 12 years. The current report represents a rare case of d-TGA longevity including multimodal assessments.**

The patient was initially diagnosed with atrial septal defect (ASD) with a cardiac murmur. The family was informed that, because of limitations of the health care system at the time, this cardiac condition was deemed inoperable, and they subsequently discontinued routine follow-up care. At the age of 37 years, the development of palpitations resulted in the patient returning for medical evaluation and care. The new-onset atrial flutter and heart failure (HF) led to the initiation of anticoagulation and diuretics; however, further evaluation was not performed. Twenty years later, emergency hospitalization was required for acute dyspnea in the night, and she was referred to our facility for worsening HF. Her oxygen saturation was 77% on reservoir mask 15 L/min, and her blood pressure was 80/55 mm Hg with atrial fibrillation at 90-110 beats per minute. The second heart sound (S2) was accentuated and single, and systolic and diastolic murmurs were auscultated at the second interspace, left sternal border. A chest radiograph disclosed bilateral hilar enlargement with a cardiothoracic ratio of 84% and bilateral pleural effusions (Supplemental Fig. S1). Laboratory data showed secondary erythrocytosis (hemoglobin level 19.8 g/dL) and an elevation of the brain natriuretic peptide level (568 pg/mL). Treatment was initiated with diuretics and respiratory support with noninvasive positive pressure ventilation; however, her saturation did not improve, necessitating further evaluation. Transthoracic echocardiography and contrast-enhanced computed tomography revealed a discordant ventriculoarterial connection with atrioventricular concordance with atrial situs solitus (Supplemental Figs. S2 and S3). She also had a 20-mm-diameter ASD with a bidirectional shunt, leading to the definitive diagnosis of dextro–transposition of the great arteries (d-TGA) with intact ventricular septum and ASD for the first time after birth. In addition, we found a giant pulmonary artery aneurysm of 74 mm in the maximum minor-axis diameter. Right heart catheterization (RHC) showed high venous pressure and severe pulmonary hypertension equivalent to systemic blood pressure with low pulmonary blood flow (Qp) and high systemic blood flow (Qs) (Table 1). Catecholamines (dobutamine 5 μg/kg/min, dopamine 3 μg/kg/min, and norepinephrine 0.3 μg/kg/min) were administered in addition to intravenous diuretics (furosemide 60 mg/d), but they did not show a favorable response, and the creatine concentration increased from 1.5 to 3.9 mg/dL without sufficient urine output. Thus, continuous hemodiafiltration and repeated pleural drainage were required to improve her respiratory distress. These therapies improved circulatory hemodynamics and respiratory status, allowing the withdrawal of continuous hemodiafiltration, and the catecholamines were successfully tapered and discontinued. A reassessment of RHC before discharge showed great improvement in central venous pressure with an increase in Qp (Supplemental Fig. S4). The patient was discharged with home oxygen therapy at 3 L/min to improve hypoxic conditions as much as possible. She was also prescribed oral medications, including enalapril 1.25 mg/d, spironolactone 25 mg/d, digoxin 0.0625 mg/d, furosemide 40 mg/d, and amiodarone 100 mg/d, for pharmacologic defibrillation for atrial fibrillation. Six months later, her rhythm returned to sinus without electrical cardioversion, and a chest radiograph showed the resolution of pleural effusion (Supplemental Fig. S5). The spirogram showed mild obstructive lung disease (Supplemental Table S1), but, together with the lung computed tomography findings, there was no apparent parenchymal lung disease. With her efforts to continue home oxygen therapy and oral medications, strict salt restriction (6 g/d), great support from a caregiver, and ongoing clinical care and management at each outpatient visit, her NYHA class Ⅲ symptoms did not worsen during regular follow-up. Repeated RHC at the age of 67 years showed further improvement in venous pressure and mean pulmonary arterial pressure, with a Qp/Qs ratio of 1.8, compared with the results a decade earlier (Supplemental Fig. S6), and there was no obvious pressure gradient in the pulmonary artery and left ventricular outflow tract. Moreover, we also confirmed that the patient’s pulmonary artery remained vasoreactive to oxygen. Cardiac magnetic resonance further confirmed a Qp/Qs ratio of 1.7 and revealed significant dilation and systolic dysfunction in the pulmonary LV (pLV), but systemic RV (sRV) function was preserved (Fig. 1, Supplemental Table S2). However, renal dysfunction, mainly due to cyanotic nephropathy, gradually progressed with age and eventually required peritoneal dialysis at the age of 69 years. Despite vigorous multidisciplinary efforts, the patient passed away a few months after starting dialysis, due to an uncontrollable massive pleural effusion.

**Table 1:** Right heart catheterization studies at the ages of 57 and 67 years

Parameter	57 years		67 years	
	On admission	Before discharge	Room air	Oxygen: 15 L/min
Rhythm	Atrial fibrillation	Atrial fibrillation	Sinus	Sinus
CVP (mm Hg)	23	10	5	3
RAP (a/v/m) (mm Hg)	–/26/24	–/12/11	10/5/5	6/3/3
RVP (s/e) (mm Hg)	99/14	92/12	108/7	98/4
LAP (a/v/m) (mm Hg)	–/26/25	–/14/14	6/5/3	6/4/3
LVP (s/e) (mm Hg)	87/18	59/11	48/3	44/2
PAP (s/d/m) (mm Hg)	90/44/62	–	50/14/27	39/14/22
PVP (a/v/m) (mm Hg)	–/34/29	–/18/17	–	–
Qp (L/min)	2.73	4.7	3.97	8.1
Qs (L/min)	6.05	4.84	2.23	1.8
Qp/Qs	0.45	0.97	1.78	4.5
PVR (WU)	12.1	–	6.0	2.3
SVR (WU)	9.6	12.0	32.7	34.4
BP (s/d/m) (mm Hg)	103/73/82	83/68/71	108/52/78	96/54/65

a, a wave; BP, blood pressure; CVP, central venous pressure; d, diastolic; e, end diastolic; LAP, left atrium pressure; LVP, left ventricle pressure; m, mean; PAP, pulmonary artery; PVP, pulmonary vein pressure; PVR, pulmonary vascular resistance; Qp, pulmonary blood flow; Qs, systemic blood flow; RAP, right atrium pressure; RVP right ventricle pressure; s, systolic; SVR, systemic vascular resistance; v, v wave; WU, Wood units.

**Figure 1:**
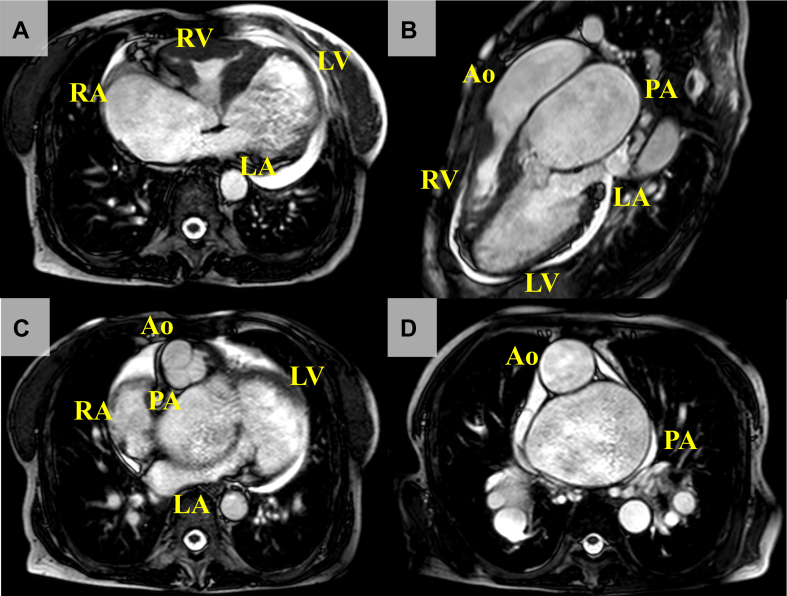
Cardiac magnetic resonance findings. (**A**) Four-chamber view, (**B**) 3-chamber view, (**C**) 5-chamber view, and (**D**) axial sectional view at the level of great arteries. Atrioventricular connection was concordant, but the aorta (Ao) originated from the anatomic right ventricle (RV) and the pulmonary artery (PA) from the anatomic left ventricle (LV), which was ventriculoarterial discordance. The Ao was seen anterior to and right of the PA, which was typical for dextro–transposition of the great arteries. A giant PA aneurysm was also recognized. LA, left atrium; RA, right atrium.

## Discussion

Unoperated history of patients with d-TGA is rare in the modern era. A report from the early 1960s provides us with the natural course of patients with uncorrected d-TGA; at that time, 89% of newborns with d-TGA died within the first year of life, and there were no survivors beyond the age of 22 years.^1^ In addition, a review of the literature demonstrated 8 unoperated d-TGA survivors into adulthood, including the current case (Supplemental Table S3). Most were female patients with ventricular septal defect, and none survived beyond the age of 60 years, with the exception of this patient. We first speculated that the optimal mixing of oxygenated and deoxygenated blood through ASD contributed greatly to her surgery-free survival for 69 years. High Qp could maintain minimal arterial oxygen saturation for survival, supported by the giant pulmonary artery aneurysm, dilated pLV, and the high value of the Qp/Qs ratio at the age of 67 years. It was certain that pulmonary vascular resistance was high, and the pulmonary circulation was severely impaired at hospitalization for HF at the age of 57 years, which would be induced by pulmonary vasoconstriction in hypoxia, but these results were obtained in the condition of acute decompensated HF. After the treatment of HF, the data of RHC completely improved and the Qp significantly increased, associated with the increase of Qp/Qs. In contrast, the persistent vasoreactivity to oxygen in this patient was curious despite the chronically elevated Qp. One possible hypothesis is that the highly oxygenated blood flow, constantly delivered to pulmonary vasculature after birth, may contribute to the prevention of pulmonary vasculature remodeling such as medial hypertrophy.^2^ Furthermore, sRV dysfunction was not evident until age 67 years, and its timing was later than pLV dysfunction. It is very rare that sRV was smaller than pLV in patients with d-TGA, but the anatomic morphology was certainly confirmed by the findings of echocardiography and cardiac magnetic resonance (Supplemental Fig. S7). We assumed that pulmonary hypertension in this patient would prevent tricuspid regurgitation in the sRV as seen in the pulmonary artery banding in congenitally corrected TGA as a palliative procedure aimed at improving the sRV function.^3^ As a result, sRV dysfunction would not progress in the early timing, and the complications associated with sRV dysfunction such as the control of ventricular arrhythmia and the exacerbation of tricuspid regurgitation were not obvious. In addition, there were no apparent complications associated with cyanosis, except for polycythemia and cyanotic nephropathy. She had no history of thrombotic or bleeding events, infectious diseases, endocrine disorders such as pheochromocytoma, or pregnancy. All of these factors have contributed to her longevity.

Although there is no consensus regarding medical therapy in patients with sRV, renin-angiotensin-aldosterone system inhibitors may be helpful in delaying sRV dysfunction.^4^ If this patient had been born in the modern era, other guideline-directed medications, such as sodium-glucose cotransporter-2 inhibitor and sacubitril/valsartan, might have provided additional benefit to her.^5^^,^^6^ Regarding surgical intervention for this patient, we have had many discussions in the multidisciplinary team. Atrial switch operation was also an option to improve her saturation; however, there is no evidence or report supporting the idea that late repair for adult patients with d-TGA improves their prognosis. Furthermore, we predicted that open heart surgery under general anesthesia would pose a high risk due to cardiac complexity, a history of HF and arrhythmia, age, and renal dysfunction. Her quality of life was not severely impaired in the uncorrected state, and we also assumed that, if she underwent surgery for d-TGA, she would inevitably require hemodialysis, which would worsen her quality of life. In addition, she did not hope for invasive intervention at that time. For these reasons, we did not choose surgical repair.

**Novel Teaching Points**

• Our report details the novel hemodynamic status and imaging findings of a patient with uncorrected d-TGA who survived to the age of 69 years.
• Even in adult patients, physicians should keep congenital heart disease in mind as a differential diagnosis of HF.
