# Supplementary material for: Uncorrected Dextro–Transposition of the Great Arteries With Intact Ventricular Septum and Atrial Septal Defect Diagnosed After Developing Heart Failure
Source: CJC Pediatr Congenit Heart Dis. 2025 Jun 27;5(1):51–4. doi: 10.1016/j.cjcpc.2025.06.003 (PMC12946901; doi:10.1016/j.cjcpc.2025.06.003)
Supplement: Supplementary Figs and Tables [file mmc1.pdf]

Supplemental Figure S1. The chest X-ray and electrocardiogram on heart failure admission at 57-years old.

(A) The electrocardiogram showed an atrial fibrillation rhythm and the rate was 90 to 110 beats per minute. Reversed R wave progression was seen. **(B)** The chest X-ray showed cardiac enlargement with a cardiothoracic ratio of 84% and bilateral pleural effusions.

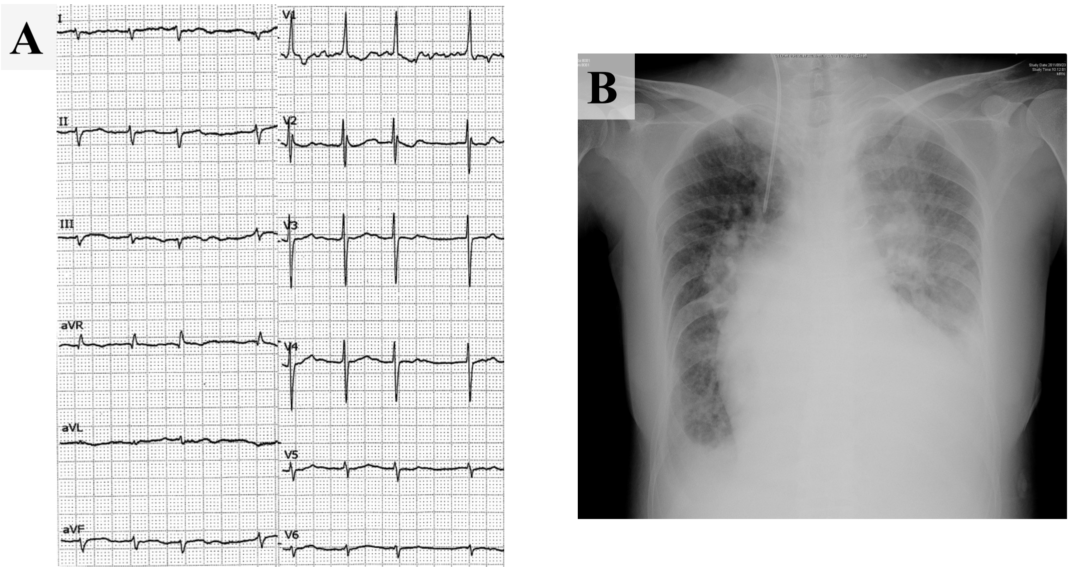

Supplemental Figure S2. The echocardiographic findings at the admission

(A) The parasternal long axis view showed the dilated pulmonary artery originated from the left ventricle with atrioventricular discordance. Left ventricle was dilated to 61mm/44mm in diastolic/systolic diameter. **(B)** In the apical four chamber view, atrial septal defect of 20mm in diameter with a bidirectional shunt was detected (red arrow).

PA, pulmonary artery; LA, left atrium; LV, left ventricle; RA, right atrium, RV, right ventricle

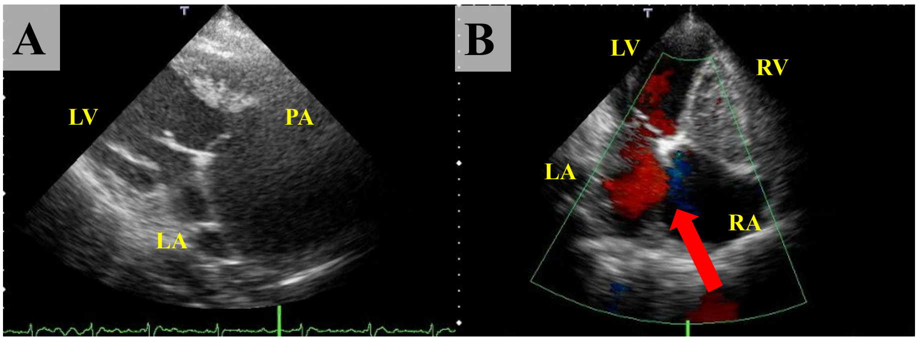

Supplemental Figure S3. The contrast computed tomography findings

(A) The images at the level of ventricular septum, **(B)** atrial septum, and **(C)** great vessels.

The systemic ventricle from which the aorta originated had significant coarse trabeculation, a more apically positioned atrioventricular valve and triangle shape, suggesting the anatomical right ventricle. Whereas, the pulmonary ventricle from which the pulmonary artery originated had the paucity of trabeculate, less apically positioned atrioventricular valve and bullet shape, whose characteristics are consistent with the anatomical left ventricle. The aorta was located right and anterior to the pulmonary artery. From these findings, the patient was diagnosed as {S, D, D} heart with ventriculoarterial discordance, usually represented as d-TGA. The atrial septal defect (red arrow) was identified, while the ventricular septum was intact. The giant pulmonary artery aneurysm of 74mm in maximum minor-axis diameter (yellow arrow) was also found.

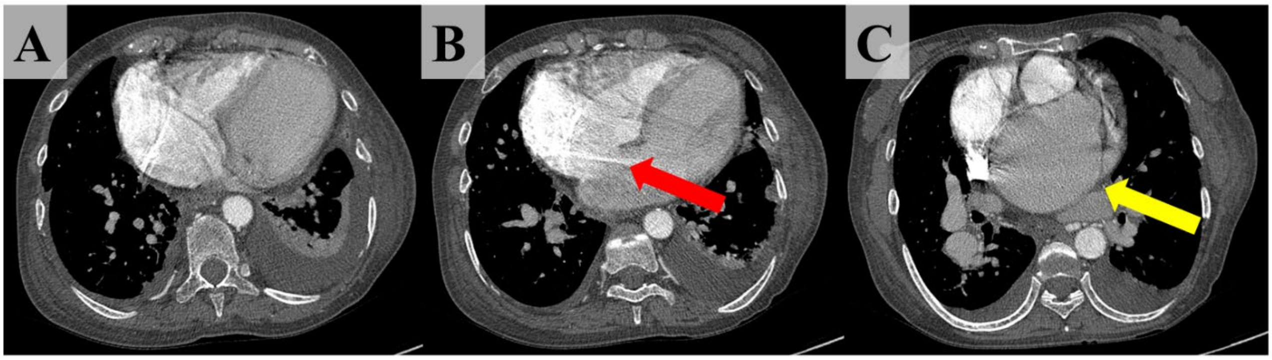

Supplemental Figure S4. The results of pressure study and oximetry run in right heart catheterization at 57-years old

(A) The pressure study revealed high venous pressure and high mean pulmonary arterial pressure, which met the criteria of pulmonary hypertension. **(B)** The data was collected in ambient air. The significant step-up in saturation between superior vena cava and the right atrium and the deterioration of saturation in the left atrium from that of the left superior pulmonary vein could support the presence of a bidirectional shunt through ASD.

SVC, superior vena cava; RA, right atrium; RV, right ventricle; LA, left atrium; LV, left ventricle; AsAo, ascending aorta; PA, pulmonary artery; LSPV, left superior pulmonary vein

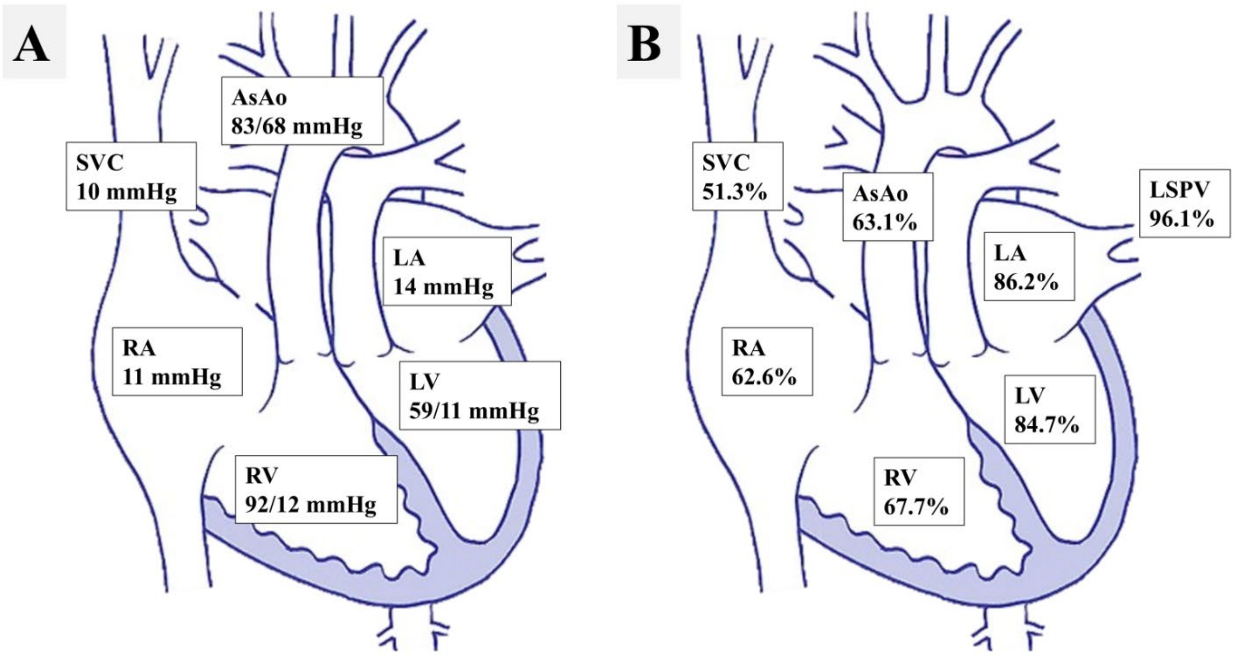

Supplemental Figure S5. The chest X ray and the electrocardiogram 6 months after discharge

(A) The electrocardiogram showed sinus rhythm with reversed R wave progression, clockwise rotation and deep S wave in lead V5,6, which implied right ventricular hypertrophy. **(B)** The chest X ray demonstrated cardiac and bilateral hilar enlargement with no plural effusion.

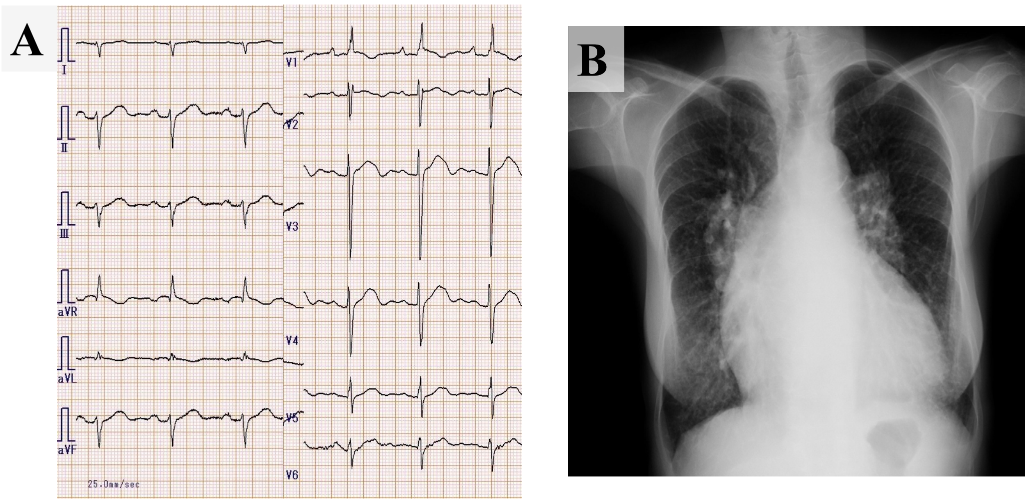

Supplemental Figure S6. The results of pressure study and oximetry run in the follow-up right heart catheterization at the age of 67

(A) The venous pressure significantly improved compared to the result of previous right heart catheterization. The patient still had pulmonary arterial hypertension; however, the mean pulmonary arterial pressure was not as elevated as before. **(B)** The oximetry run revealed the mixing of oxygenated and deoxygenate blood via atrial septal defect, and the calculated Qp/Qs ratio was 1.78.

RA, right atrium; RV, right ventricle; LA, left atrium; LV, left ventricle; AsAo, ascending aorta; PA, pulmonary artery; LSPV, left pulmonary artery wedge.

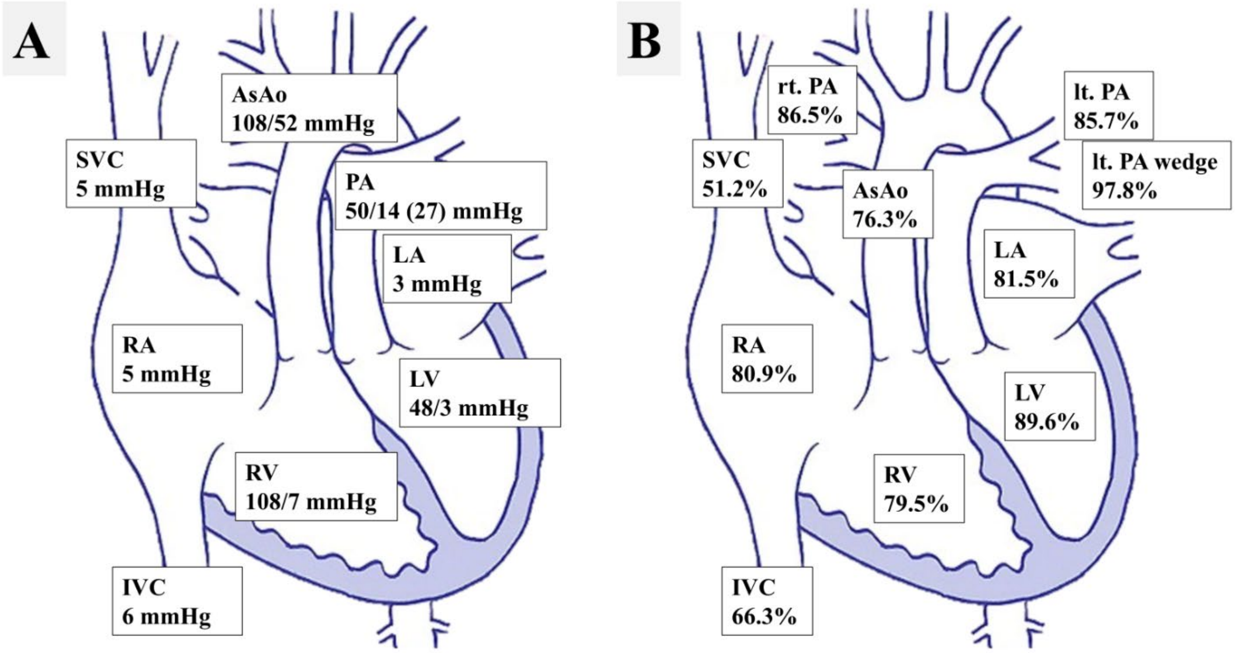

Supplemental Figure S7. Segmental assessments of the ventricles in this patient

The images of echocardiography and CMR study showed that a ventricular chamber which connected to the aorta comprised a moderator band **(A, B)**, coarse trabeculations **(C)** and three components (inlet, apical and outlet portions) **(D)**. It was also found to be a crescentic shape **(C)**. In this chamber, atrioventricular valve was more apically inserted **(E)**. Furthermore, the connection between this chamber and the great vessel included a complete ring of muscle, corresponding to the conal septum **(F)**. These findings supported that this chamber was anatomically the right ventricle.

In contrast, the other ventricle chamber had fibrous continuity of the atrioventricular valve and the great vessel **(G)**. The papillary muscles in this chamber were not attached to the septum **(H)**, and the shape of this chamber was more elliptical. From these findings, we thought that this chamber was morphologically the left ventricle.

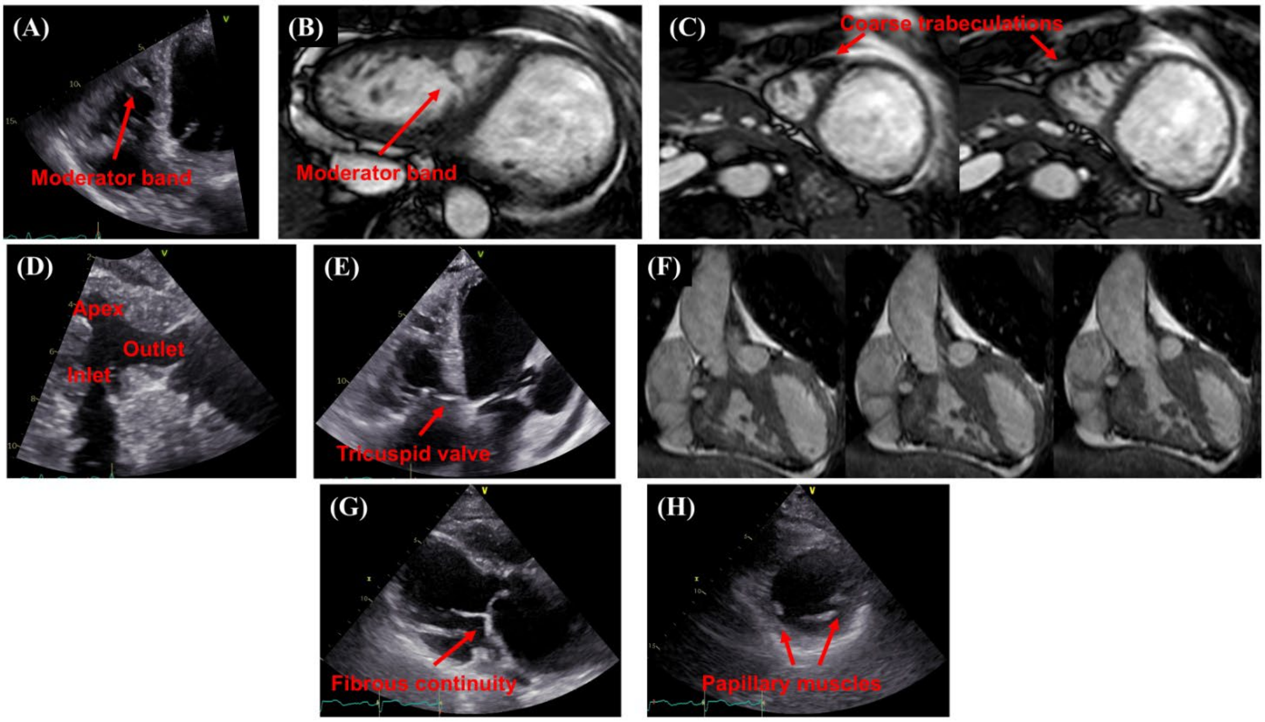

Supplemental Table S1. Spirometry finding

Parameter	
%VC	88.5%
FEV 1/FVC	69.6%
DLco	13.91 mL/min/mmHg (99.1%)
DLco/VA	5.0 mL/min/mmHg (104.9%)

%VC, Vital capacity as percent of predicted.

FEV 1/FVC, Forced expiratory volume in one second / Forced vital capacity

DLco, Diffusion capacity of the lungs for carbon monoxide (Also known as transfer factor for carbon monoxide or TLCO)

DLco/VA, DLco divided by the alveolar volume

Supplemental Table S2. Cardiac magnetic resonance finding

Parameter	
RVEDV (ml)	91.7 (78% of normal)
RVESV (ml)	28.1
RVEF (%)	69.3
LVEDV (ml)	196.5 (184% of normal)
LVESV (ml)	105.3
LVEF (%)	46.4
Qp (L/min)	4.8
Qs (L/min)	2.8
Qp/Qs	1.7
PR regurgitation fraction (%)	11.5
TR regurgitation fraction (%)	35.7

RVEDV, right ventricle end diastolic volume; RVESV, right ventricle end systolic volume; RVEF, right ventricle ejection fraction; LVEDV, left ventricle end diastolic volume; LVESV, left ventricle end systolic volume; LVEF, left ventricle ejection fraction; Qp, pulmonary blood flow; Qs, systemic blood flow; PR, pulmonary regurgitation; TR, tricuspid regurgitation.

Supplemental Table S3. Previous reports of untreated d-TGA survivors

	Age	Sex	Complications	Age of developing HF	Age of the diagnosis	Reference
Case1	29	Female	VSD	Unknown	Unknown	Shaher RM et al. *Br Heart J.* 1963;25:211-218
Case2	31	Female	ASD, VSD	27	31	Oladapo O et al. *Nigerian Journal of Cardiology.* 2016;13:86-89
Case3	33	Female	ASD, VSD, PS	20	23	Narcisse-Alvarez JF et al. *Cardiol Young.* 2019;29:720-724
Case4	38	Female	PFO, VSD	38	Autopsy	Messeloff CR et al. *Am Heart J.* 1951;42:467-471
Case5	40	Female	ASD, VSD	40	40	Naganur SH et al. *Ann Pediatr Cardiol.* 2020;13:72-74
Case6	56	Unknown	Unknown	Unknown	Unknown	Nichol AD et al. *J Am Med Assoc.* 1951;147:645-648
Case7	58	Female	ASD	Unknown	58	Gallego P et al. *Pediatr Cardiol.* 1998;19:358-360
Case8 (Current case)	69	Female	ASD	37	57	

ASD, atrial septal defect; VSD, ventricular septal defect; PS, pulmonary stenosis; PFO, patent foramen ovale; HF, heart failure
